# Administration of antibiotics via the respiratory tract for the prevention of ICU-acquired pneumonia: a meta-analysis of comparative trials

**DOI:** 10.1186/cc5032

**Published:** 2006-08-25

**Authors:** Matthew E Falagas, Ilias I Siempos, Ioannis A Bliziotis, Argyris Michalopoulos

**Affiliations:** 1 Alfa Institute of Biomedical Sciences (AIBS), Athens, Greece; 2 Department of Medicine, Henry Dunant Hospital, Athens, Greece; 3 Department of Medicine, Tufts University School of Medicine, Boston, Massachusetts, USA; 4 Intensive Care Unit, Henry Dunant Hospital, Athens, Greece

## Abstract

**Introduction:**

The administration of prophylactic antibiotics via the respiratory tract is one of several strategies for the prevention of intensive care unit (ICU)-acquired pneumonia. We systematically examined the available evidence regarding the effect of prophylactic antibiotics administered via the respiratory tract on the development of ICU-acquired pneumonia, mortality, colonization of the respiratory tract, emergence of antimicrobial resistance, and toxicity.

**Methods:**

We searched the PubMed database (January 1950 to September 2005) and references from relevant articles to identify trials that provided comparative data regarding the above-mentioned outcomes. Two investigators independently performed the data extraction to calculate the effect of the studied intervention on clinically relevant outcomes.

**Results:**

Our meta-analysis includes 8 comparative trials (5 randomized controlled trials (RCTs) and 3 non-randomized trials) studying gentamicin (3 trials), polymyxins (3 trials), tobramycin (1 trial), and ceftazidime (1 trial) that studied 1,877 patients. Our primary analysis, which included the 5 RCTs, revealed that ICU-acquired pneumonia was less common in the group of patients that received the antibiotic prophylaxis (odds ratio (OR) = 0.49, 95% confidence interval (CI) 0.32–0.76). No difference in mortality was found between the compared groups (OR = 0.86, 95% CI 0.55–1.32). Data were too limited to permit an analysis of colonization with *Pseudomonas aeruginosa*. A secondary analysis, adding the three non-randomized comparative trials, did not reveal substantially different results regarding ICU-acquired pneumonia and mortality, while fewer patients were colonized with *P. aeruginosa *in the group that received prophylaxis, compared to the group of patients that received no prophylaxis (OR = 0.51, 95% CI 0.30–0.86). No serious drug-related toxicity was noted. No meaningful systematic analysis of the evidence regarding the emergence of resistance could be performed in the studies included in our meta-analysis.

**Conclusion:**

The limited available evidence supports that prophylactic administration of antibiotics via the respiratory tract is associated with reduction of occurrence of ICU-acquired pneumonia. However, there is evidence from non-comparative studies that this preventive strategy may lead to an increase in the emergence of resistant bacteria. Thus, further investigation, at least in ICU patients at high risk for development of ICU-acquired pneumonia, is warranted, including a more systematic evaluation of issues related to the emergence of resistance.

## Introduction

Intensive care unit (ICU)-acquired infection of the respiratory tract is a common complication among patients who receive medical care in this setting. Colonization of the respiratory tract by Gram-negative and Gram-positive bacteria may precede infection of the lower respiratory tract, including pneumonia, that is associated with considerable morbidity and mortality. There have been several efforts to reduce the development of ICU-acquired pneumonia using various strategies, including selective bowel decontamination, that have been summarized recently [1,2]. Among them are studies examining the effectiveness of administration of antimicrobial agents via the respiratory tract in the prevention of bacterial colonization of the respiratory tract and ICU-acquired pneumonia.

Recommendations from the Centers for Disease Control and Prevention strongly discourage the administration of antibiotics via the respiratory tract for the prevention of ICU-acquired pneumonia [3,4]. In addition, the Canadian Critical Care Trials Group and the Canadian Critical Care Society also discourage such a strategy in the published clinical guidelines regarding the evidence-based clinical practice for the prevention of ventilator-associated pneumonia [1]. We sought to systematically examine the evidence related to the above guidelines by performing a meta-analysis of comparative trials studying the effect of the administration of antibiotics via the respiratory tract on the colonization of the respiratory tract by bacteria and development of ICU-acquired pneumonia.

## Methods

### Data sources

Two investigators (IIS and IAB) independently performed the literature search, study selection, and data extraction. Discrepancies between these two investigators were resolved in meetings of all authors. The relevant comparative trials for this meta-analysis were identified from searches of PubMed (January 1950 to September 2005) and references from relevant articles. The key terms that we used for the literature search were aerosolised, nebulised, nebulized, endotracheal, intratracheal, micronebulised, micronebulized, nosocomial pneumonia, ventilator-associated pneumonia, and ICU-acquired pneumonia. Abstracts presented in international conferences were not searched.

### Study selection

A comparative trial was considered eligible for inclusion in our meta-analysis if it compared the effectiveness of an antibiotic administered via the respiratory tract with placebo or no drug on the colonization of the respiratory tract, ICU-acquired pneumonia, and/or mortality. Both randomized controlled trials (RCTs) and non-randomized comparative trials were allowed to be included in our meta-analysis. Articles written in any language were allowed to be included in our meta-analysis.

### Data extraction

The data extracted from the articles for further analysis were the study population, the dosage and the duration of the administered drugs, the number of clinically evaluable patients, ICU-acquired pneumonia, colonization of the respiratory tract by various micro-organisms, mortality, emergence of resistance, and toxicity. A quality review of each RCT was performed by examining details of randomization, generation of random numbers, details of double-blinding procedure, information on withdrawals, and concealment of allocation [5]. One point was awarded for the specification of each of the above criteria; the maximum score for a study is 5. High quality RCTs score more than 2 points, while low quality RCTs score 2 or fewer points, according to the reported methodology.

### Definition of outcomes

The occurrence of pneumonia during the ICU stay and all cause and pneumonia-related mortality were considered the primary outcome measures of this meta-analysis. In addition, colonization with *Pseudomonas aeruginosa*, any reported toxicity, and emergence of resistance were considered secondary outcomes of analysis. Pneumonia was defined by clinical, laboratory, and/or imaging findings attributed by the authors of the trials to this infection. However, if the cases of pneumonia were reported separately into possible, probable, or definitive (documented), only the last two categories were included in our analysis. Colonization was defined by the isolation of one or more micro-organisms from sputum, bronchial secretions, or bronchoalveolar lavage specimens of the patients without accompanying evidence of infection of the respiratory tract. Any toxicity or emergence of antimicrobial resistance reported by the authors of the included studies was evaluated and analyzed when possible.

We performed a primary analysis of outcomes by including only RCTs. In addition, we performed secondary analyses by including all trials (both RCTs and non-randomized comparative trials), as well as by examining outcomes in subsets of patients, namely, intubated patients, patients treated with polymyxins, patients that received prophylactic antibiotics in aerosolized form, and patients in whom prophylactic antibiotics were instilled endotracheally.

### Data analysis and statistical methods

Statistical analyses were performed using the 'Meta-analyst' software (Joseph Lau, Tufts University School of Medicine, Boston, MA, USA) and the S-Plus 6.1 statistical software (Insightful Corp., Seattle, WA, USA). Pooled odds ratios (ORs) and 95% confidence intervals (CI) for all primary and secondary outcomes were calculated by using both the Mantel-Haenszel fixed effects and the DerSimonian-Laird random effects models [6-8]. The heterogeneity between studies was assessed by using the chi-square test; a *p* value lower than 0.10 was defined to note statistical significance in the analysis of heterogeneity. For all analyses, results from the fixed effects model are presented only when there was no heterogeneity between studies; otherwise results from the random effects model are presented. The reported outcome rates of the analyzed studies were weighted by the inverse of their variance with the fixed effects model. Small studies bias was assessed by the funnel plot method using the Egger's test [9].

## Results

### Study selection

**Figure 1:**
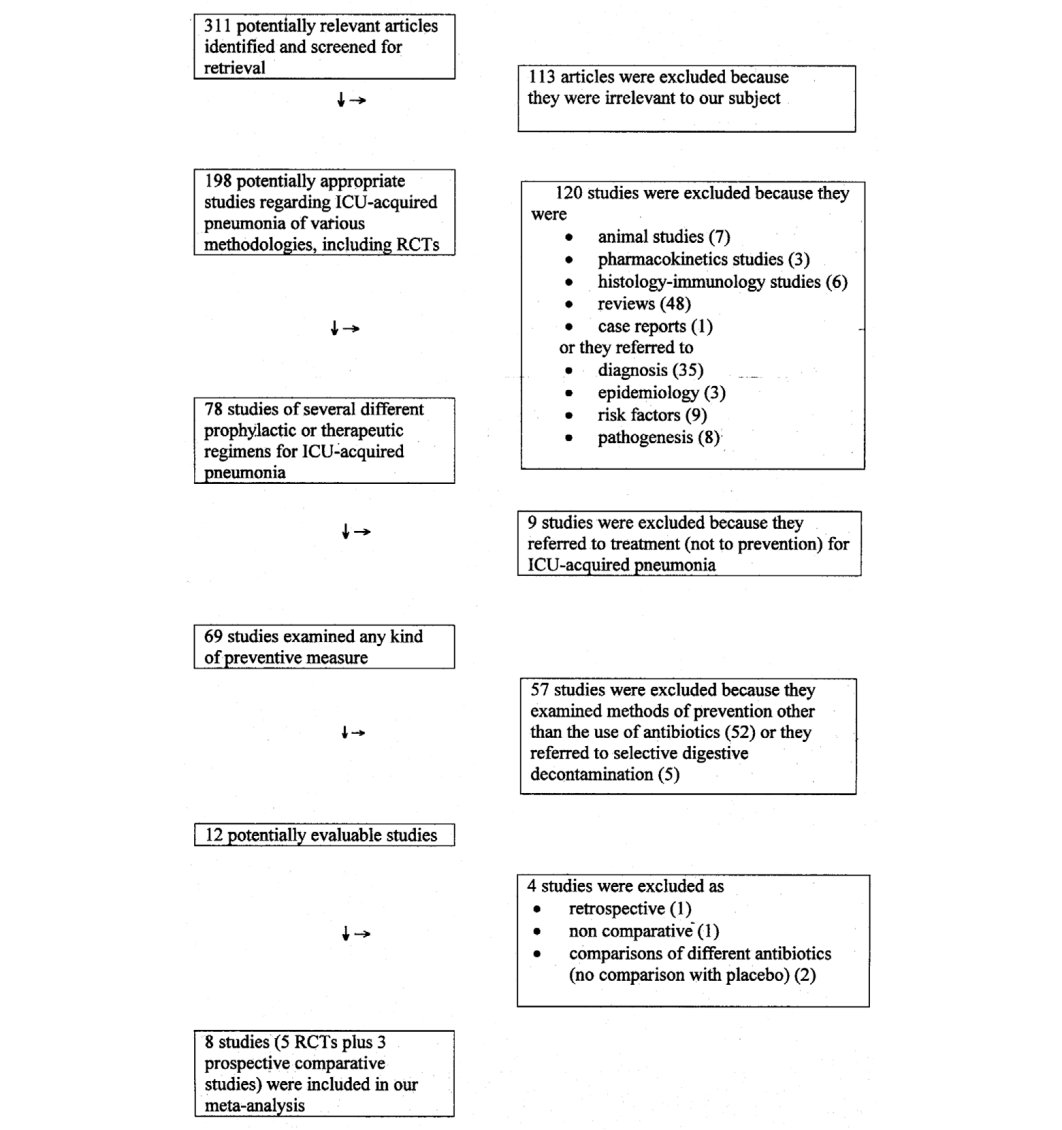
Flow diagram of reviewed articles.

In Figure 1 we present the steps we followed in order to select the relevant studies for our analysis. As shown, we identified 311 studies from the search of the PubMed database, as well as from the reading of the references of relevant studies. From these, we identified 12 studies that examined the use of prophylactic antibiotics administered via the respiratory tract for the prevention of ICU-acquired pneumonia [10-21]. Finally, eight studies (five RCTs plus three non-randomized prospective trials) that compared the administration of prophylactic antibiotics via the respiratory tract with the administration of placebo (five studies) or no drug (three studies) fulfilled our inclusion criteria and were further analyzed (Table 1) [11,13,15,17-21]. The eight studies encompassed a total of 1,877 patients.

The quality assessment of the five RCTs included in our study (evaluating the presence of randomization and blinding, their appropriateness, and the presence of information on withdrawals) showed that the quality of two RCTs was high [13,21], while the quality of the other three was low (equal to or less than two points) [11,17,18]. The mean quality score of the included RCTs was 2.6 (in a 0 to 5 scale), which is considered good.

### Drug administration

In Table 1 we present various characteristics of the trials included in our analysis. In four of the analyzed studies the antibiotic prophylaxis was given in the form of aerosolized preparations [11,15,18,21] whereas antibiotics were administered with endotracheal instillation to patients in the rest of the studies [13,17,19,20]. The drugs used were gentamicin (three studies) [13,17,20], polymyxins (three studies; specifically, polymyxin B in two studies [11,15] and colistin in one study [19]), tobramycin (one study) [18], and ceftazidime (one study) [21]. The duration of therapy was one week in one study, two weeks in two studies, until the time of extubation in two studies, and throughout the entire ICU stay of patients in the three remaining studies.

Only two of the trials included in our analysis provided data regarding the pulmonary drug concentrations of the drugs administered via the respiratory tract. In the first trial [21], in which ceftazidime was administered by aerosol, ceftazidime concentrations were detectable by bronchoalveolar lavage procedures in 16 of 19 ceftazidime-recipients; 3 of these 16 patients had concentrations below the breakpoint for ceftazidime sensitivity. In the second trial gentamicin was instilled endotracheally, the mean level of which in bronchial secretions was 230 μg/ml ± 72 μg/ml. Thus, the scarcity of relevant data did not allow us to validate the effectiveness of the various modes of administration via the respiratory tract [13].

Data regarding the administration of systemic antibiotics during the administration of prophylactic antibiotics via the respiratory tract was reported in five of the analyzed studies; however, no pooling of data could be performed since there was considerable heterogeneity [11,13,15,18,21]. Specifically, Klastersky and colleagues [13] reported that systemic antibiotics were given more frequently (*p* < 0.01) to the patients in the placebo-treated group than those who were treated with gentamicin endotracheally. In the study by Rathgeber and colleagues [18], it is mentioned that the subgroup of patients with multiple traumas received systemic prophylaxis with metronidazole and cefuroxime, regardless of their randomization to receive prophylaxis or not via the respiratory tract. Greenfield and colleagues [11] reported that 88% of the polymyxin-treated patients and 76% of the patients in the placebo group received antibiotics systemically during their ICU stay (which was also the time during which they received aerosolized polymyxin B or placebo). Similarly, in the study by Klick and colleagues [15], 53% of the polymyxin-treated patients and 49% of the patients in the placebo group received antibiotics systemically. Finally, in the study by Wood and colleagues [21], only data regarding patients that developed pneumonia were presented; systemic antibiotics had been administered in 6/6 patients in the ceftazidime group and 11/13 in the control group, a result without statistical significance.

### Mortality

**Figure 2:**
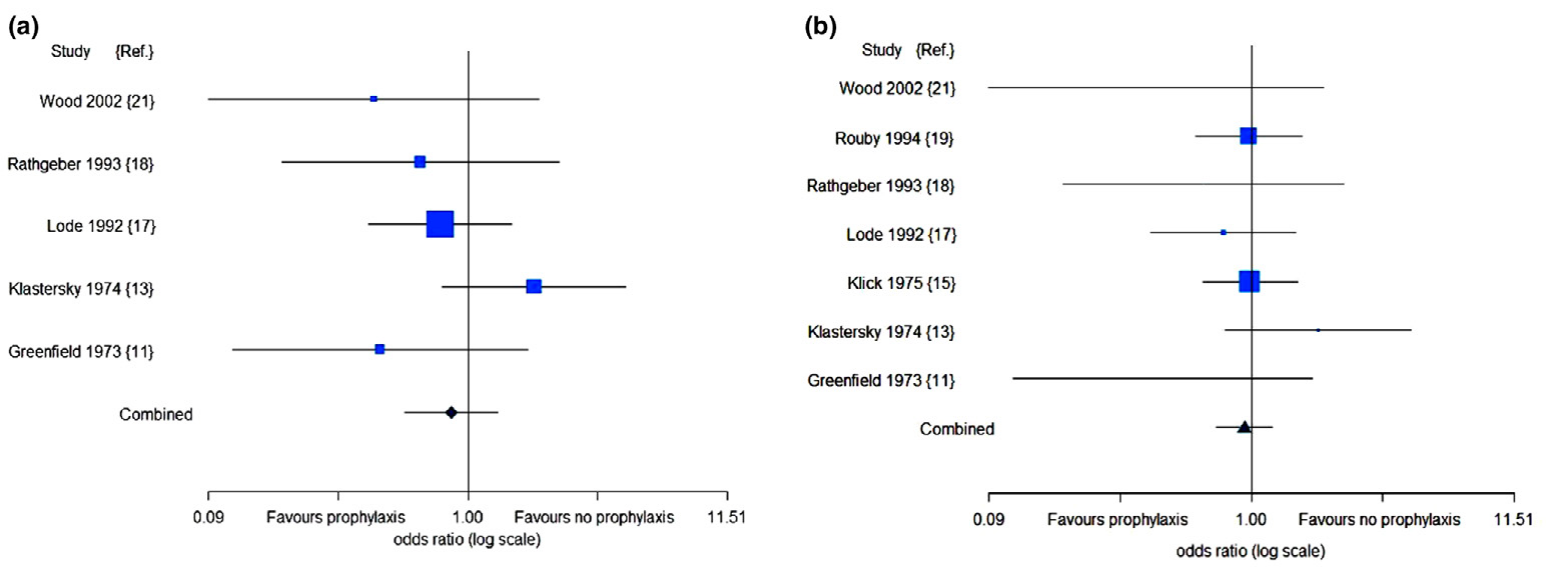
Odds ratios of mortality between patients who received antibiotic prophylaxis via the respiratory tract and those who received placebo or no therapy. **(a) **Primary analysis (only randomized controlled trials); **(b) **secondary analysis (including non-randomized trials). Vertical line = 'no difference' point in mortality between the two regimens. Horizontal lines = 95% confidence interval. Square = odds ratio; the size of each square denotes the proportion of information given by each trial. Diamond/triangle = pooled odds ratio for all studies.

In Table 2 we present data regarding the outcomes of our analysis. All cause mortality during the ICU stay was reported in all five included RCTs (Table 2) [11,13,17,18,21]. No difference in mortality between prophylactic antibiotic therapy administered via the respiratory tract and no therapy or placebo therapy was found (all cause mortality; OR = 0.86, 95% CI 0.55–1.32, fixed effects model; Figure 2a).

Pneumonia-related mortality was reported in two RCTs (Table 2) [13,18]. In each of these RCTs no difference in pneumonia-related mortality was found between patients in the prophylactic antibiotic therapy group and in the control group (pneumonia-related mortality: 1st RCT [13], 2/43 (5%) versus 4/42 (10%), *p* = 0.4; 2nd RCT [18], 2/29 (7%) versus 4/40 (10%), *p* = 0.99).

### ICU-acquired pneumonia

**Figure 3:**
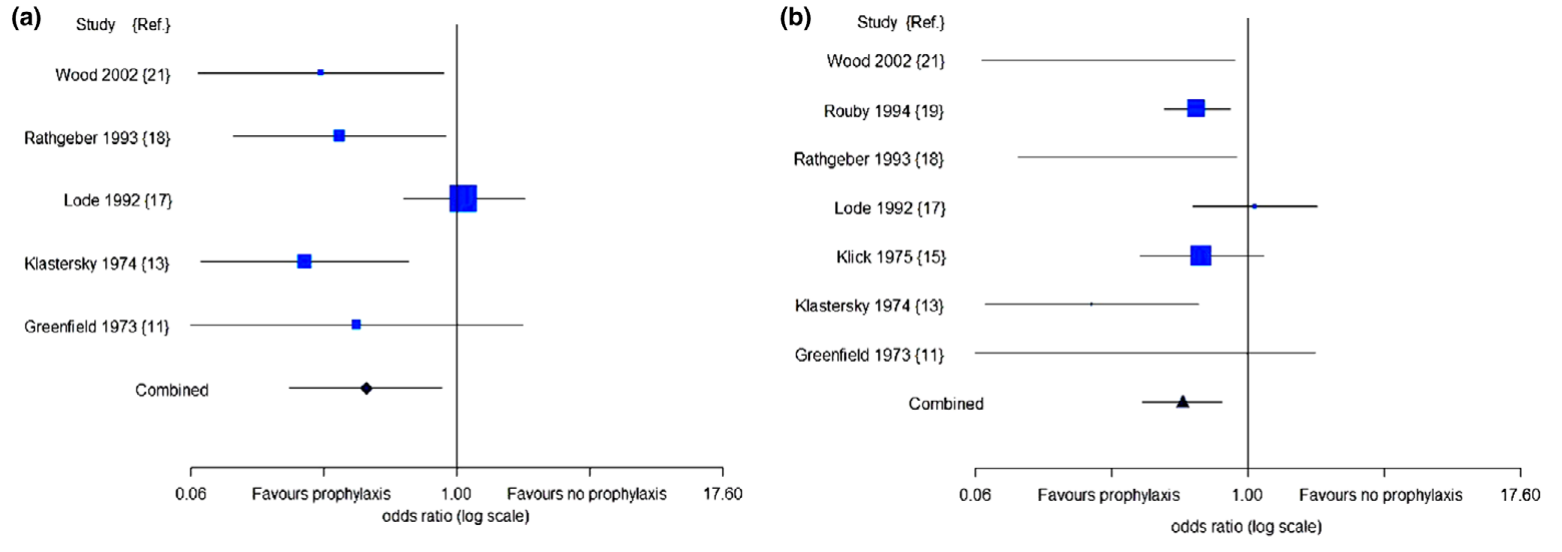
Odds ratios of intensive care unit-acquired pneumonia between patients who received antibiotic prophylaxis via the respiratory tract and those who received placebo or no therapy. **(a) **Primary analysis (only randomized controlled trials); **(b) **secondary analysis (including non-randomized trials). Vertical line = 'no difference' point in intensive care unit-acquired pneumonia between the two regimens. Horizontal lines = 95% confidence interval. Square = odds ratio; the size of each square denotes the proportion of information given by each trial. Diamond/triangle = pooled odds ratio for all studies.

Pneumonia occurred less frequently in the prophylaxis arm compared to the no-prophylaxis arm, a statistically significant result (ICU-acquired pneumonia: OR = 0.49, 95% CI 0.32–0.76, fixed effects model, 5 RCTs; Figure 3a) [11,13,17,18,21].

### Colonization with *P. aeruginosa*

Four RCTs reported specific data regarding the colonization of the respiratory tract by *P. aeruginosa *[11,13,17,18]. However, two of them reported only the proportion of *P. aeruginosa *isolates among all isolated organisms without specifically referring to the number of patients from whom these organisms were isolated [13,18]. Thus, data from the remaining two RCTs [11,17] were not enough to permit a meta-analysis of colonization with *P. aeruginosa*. In each of these RCTs [11,17] a similar proportion of patients was colonized with *P. aeruginosa *in the group that received prophylaxis, compared to the group of patients that received no prophylaxis (colonization with *P. aeruginosa*: 1st RCT [11], 0/33 (0%) versus 3/25 (12%), *p* = 0.07; 2nd RCT [18], 2/85 (2%) versus 6/77 (8%), *p* = 0.15).

### Emergence of resistance

Data regarding the number and type of the isolated organisms were reported in six of the studies (three RCTs [13,18,21]) included in our analysis [13,15,17,18,20,21]. However, there was limited information regarding the *in vitro *antimicrobial susceptibility of the isolated pathogens. Specifically, data regarding bacteria resistant to gentamicin, polymyxins, and ceftazidime were reported in one [13], three [11,15,19] and one [21] study, respectively. Unfortunately, no systematic analysis of the emergence of resistance could be performed in the studies included in our meta-analysis to allow a meaningful synthesis of evidence regarding this important outcome. In Table 2 we present the information regarding the emergence of resistance reported in the analyzed studies, if any.

### Toxicity

In five of the included studies no data regarding toxicity were reported. In two RCTs it was reported that no toxicity was observed during the trials [18,21], whereas in the remaining RCT the authors characterized the observed toxicity negligible [11], without reporting any further detail (Table 2).

### Secondary analyses

The ICU-acquired pneumonia, all cause mortality, pneumonia-related mortality, and colonization with *P. aeruginosa *were analyzed by also including the three non-randomized comparative trials [15,19,20]: pneumonia, OR = 0.50, 95% CI 0.33–0.76, data from 7 studies [11,13,15,17-19,21] (Figure 3b); mortality, OR = 0.93, 95% CI 0.72–1.22, data from 7 studies [11,13,15,17-19,21] (Figure 2b); pneumonia-related mortality, OR = 0.98, 95% CI 0.39–2.49, fixed effects model, data from 3 studies; colonization with *P. aeruginosa*, OR = 0.51, 95% CI 0.30–0.86, data from 4 studies [11,15,17,20]. Of note, the study by Klick and colleagues [15] was terminated prematurely because of an increase in colonization and infection by *P. aeruginosa *in the group without prophylaxis, which forced the physicians to use prophylaxis with aerosolized polymyxin for all patients due to the good results that were observed with this mode of treatment in their unit [15].

In addition, ICU-acquired pneumonia and all cause mortality were analyzed in four subsets of patients. The 1st subset comprised studies that included only intubated patients (pneumonia, OR = 0.60, 95% CI 0.45–0.80; and mortality, OR = 0.83, 95% CI 0.58–1.21; 4 studies analyzed for both outcomes [17-19,21]); these studies also represented the subset of the most recent studies, published after 1990. The 2nd subset comprised studies that examined polymyxins as antibiotic prophylaxis (pneumonia, OR = 0.58, 95% CI 0.43–0.79; mortality, OR = 0.94, 95% CI 0.68–1.30; 3 studies analyzed for both outcomes [11,15,19]). The 3rd subset comprised studies in which aerosolized prophylactic antibiotics were administered (pneumonia, OR = 0.44, 95% CI 0.27–0.72; mortality, OR = 0.84, 95% CI 0.57–1.24; 4 studies analyzed for both outcomes [11,15,18,21]). The 4th subset comprised studies in which prophylactic antibiotics were instilled endotracheally (pneumonia, OR = 0.61, 95% CI 0.45–0.81; mortality, OR = 1.04, 95% CI 0.68–1.59; 3 studies analyzed for both outcomes [13,17,19]).

## Discussion

The main finding of our study is that development of ICU-acquired pneumonia is less common in patients who received prophylactic antibiotics via the respiratory tract compared to placebo or no drug. Specifically, the OR for development of ICU-acquired pneumonia was 0.50 for patients who received antibiotic prophylaxis via the respiratory tract compared to those who received no prophylaxis. No difference in mortality was found between patients in the two compared groups. Data from RCTs were not enough to permit an analysis of colonization with *P. aeruginosa*. Nevertheless, in a secondary analysis that also included the three non-randomized trials, colonization with *P. aeruginosa *was found to be less in the group of patients that received prophylaxis. To our knowledge, this is the first meta-analysis that has examined the effectiveness of prophylactic antibiotics administered via the respiratory tract against the development of ICU-acquired pneumonia.

Some data from animal and laboratory studies support the prophylactic use of antibiotics administered locally in the respiratory tract [22-24]. Animal studies have provided supporting data for the local administration of antibiotics for the prevention of development of colonization and infection of the respiratory tract. Specifically, prevention of colonization of the respiratory tract by highly invasive micro-organisms was shown after the prophylactic administration of topical instillation of polymyxin B into the respiratory tract in 13 consecutive studied baboons [22].

In addition, pharmacokinetic studies showed that the concentration in the endobronchial fluid of antibiotics administered via the respiratory tract is high. Specifically, in a comparative study of the administration of 2 mg/kg of body weight of gentamicin via the intramuscular route or the respiratory tract showed that, after systemic administration, the serum concentration of gentamicin was more than 6 μg/ml and the endobronchial less than 2 μg/ml, while the respective values after endotracheal instillation of the antibiotic were 1 μg/ml and 400 μg/ml [24]. In another study of lung distribution bronchokinetics of aerosolized tobramycin, the mean lung tissue concentrations of tobramycin were 5.5 and 3.61 μg/ml 4 and 12 hours after nebulization, respectively [23]. It should be emphasized that the effect of the specific way of administration of antibiotics via the respiratory tract on the concentrations accomplished in the endobronchial fluid or the lung parenchyma has not been systematically examined. For example, Wood and colleagues [25] reported that the amount of the nebulized dose that reaches the distal airways of the lungs may be several times higher with the use of an appropriate nebulizer, ventilator and administration technique compared to non-standardized ways of administration of antibiotics into the respiratory tract.

In addition to patients who receive care in the ICU setting, patients susceptible to colonization of the respiratory tract by various bacteria and, subsequently, the development of lower respiratory tract infections are those with underlying lung disease, including cystic fibrosis, bronchiectasis, and severe chronic obstructive pulmonary diseases. The effect of the administration of antibiotics via the respiratory tract on the prevention of respiratory tract colonization and infection was also investigated in these patient populations. It has been shown that the bacteria most frequently isolated from the sputum of patients with bronchiectasis are *P. aeruginosa*, *Staphylococcus aureus*, *Haemophilus influenzae*, and *Streptococcus pneumoniae*. It has also been shown that an increase of *P. aeruginosa *local density in the respiratory tract may be associated with deterioration of lung function and increase of morbidity and mortality of patients with cystic fibrosis. Only three RCTs have examined the prophylactic effect of antibiotics administered via the respiratory tract in patients with bronchiectasis [26-28]. In general, a reduction of the colonization and infection of the respiratory tract was noted in these trials, although concerns about possible development of antimicrobial resistance were also raised.

The Canadian Critical Care Trials Group and the Canadian Critical Care Society [1] as well as the Centers for Disease Control and Prevention [3,4] suggest the avoidance of the prophylactic administration of antibiotics via the respiratory tract because of concerns about development of resistant pathogens as well as the toxicity related to the administered agents, based mainly on data from non-comparative trials [29-32]. For example, in an old non-comparative study, colonization of the respiratory tract by bacteria resistant to polymyxins, such as S. *aureus*, coagulase-negative staphylococci, *Enterococcus *spp., flavobacteria, *Serratia *spp., *Proteus *spp. as well as *Candida *spp., was noted in a proportion of patients who received prophylactic polymyxin B via the respiratory tract [10]. Although the findings of that study indicated that the administration of polymyxin via the respiratory tract for the prevention of ICU pneumonia was not effective and was in fact harmful because it was associated with toxicity and emergence of resistance, no direct comparison was made in that study with a group of patients that did not receive such a preventive therapy. Also, the authors of that study found an increase in pneumonia-associated mortality during the use of aerosolized polymyxin, compared to previous time periods in the same center when no polymyxin via the respiratory tract was used, a fact thought to be related to the emergence of the aforementioned organisms. However, the authors did not perform statistical comparisons to evaluate this difference and it should be emphasized that they compared patients from different time periods.

The emergence of resistant strains after the use of inhaled polymyxins has also been reported in another non-comparative study. In that study [33] an outbreak of nosocomial *Flavobacterium meningosepticum *respiratory infections was considered to be associated with prophylactic use of aerosolized polymyxin B. Twenty isolates of *F. meningosepticum *were isolated from nine patients during a two and a half month period. In five of them the bacterium caused pneumonia, resulting in two deaths. All isolates were ciprofloxacin-only susceptible. In addition, in the study by Klastersky and colleagues [14], the comparison of two prophylactic aerosolized regimens, namely gentamicin and aminosidin-polymyxin B combination, showed that the use of these regimens, and especially the first one, was associated with the emergence of gentamicin-resistant strains.

The limited available evidence from the eight comparative trials that we analyzed does not directly support the concern for the development of resistant pathogens, as it was reported in the four aforementioned studies. A possible explanation for this is that, in the included studies, and especially in the more recent ones [18,19,21], the prophylactic antibiotics were administered for shorter periods of time compared to the studies discussed above. Also, the emergence of resistant organisms in the studies included in our meta-analysis, apart from being rare, was not found to be associated with any form of morbidity or with increased mortality. It should be emphasized that the decrease in the proportion of patients that develop pneumonia should also result in a substantial decrease in the overall use of systemically administered antibiotics. This in turn may lead to a decrease in the emergence of organisms with antimicrobial resistance. However, data regarding this issue from the analyzed studies were too heterogeneous to make any meaningful synthesis of them. In fact, as none of the studies included in our meta-analysis looked systematically at emergence of resistance, we cannot comment on whether or not administration of topical antimicrobial agents is associated with development of resistance.

It is noteworthy that no major toxicity of the antibiotics administered via the respiratory tract as prophylaxis was noted in any of the patients included in the analyzed trials that reported relevant data. However, it should also be noted that local adverse effects from the respiratory tract after the prophylactic or therapeutic administration of antibiotics were reported in other studies. Most of these, however, were related to minor or moderate bronchospasm that was alleviated by the appropriate bronchodilator treatment [34,35].

Our study has several limitations. First, we included trials performed in different time periods; this fact has an effect on the antimicrobial resistance pattern of the isolated pathogens in different studies and methods of diagnosis of pneumonia. For example, the very small proportion of methicillin-resistant staphylococci isolated in most of the analyzed studies represents a significant difference in comparison to the current situation in most ICUs worldwide. Second, we included trials that examined different medications; however, we performed sensitivity analysis for a specific class of antibiotics, namely polymyxins, administered via the respiratory tract and we found that the results regarding the positive effect of the prophylactic local agents on the development of ICU-acquired pneumonia and overall mortality were not different from those of the main analysis. Third, we analyzed data mainly from patients who were receiving mechanical ventilation, although three studies included a minority of patients who were receiving care at the ICU setting but not mechanical ventilation. Again, sensitivity analysis of the studies that included only patients with mechanical ventilation did not reveal different results compared to the main analyses regarding the primary outcomes of analysis. Fourth, we included in our meta-analysis trials that were performed on populations that had a different profile of risk factors. Fifth, we analyzed only the effect of antibiotic prophylaxis via the respiratory tract on colonization by *P. aeruginosa *due to the unavailability of relevant data for other organisms. Sixth, the change from a positive to a negative culture of tracheobronchial secretion specimens with the administration of topical antibiotics may be due to suppression of microbial growth rather than true eradication of colonization. However, even if this change is due to suppression of microbial growth, it may be of value as it is associated with reduction of occurrence of negative outcomes [36].

Another limitation of our meta-analysis is that the effect of prophylactic antibiotics administered via the respiratory tract on the length of the ICU stay and the hospital stay was not systematically analyzed in the included trials. In addition, the studies that were included in our meta-analysis did not report any data regarding the cost effectiveness of the administration of antibiotics via the respiratory tract for the prevention of ICU-acquired pneumonia. Furthermore, we should note that there may be a placebo effect, that is, that the administration of placebo, which is usually a small amount of normal saline in an aerosolized form, may have an effect on the colonization and, subsequently, the infection of the respiratory tract [37]. Also, currently recommended strategies for reduction of ICU pneumonia, such as ventilator circuit changes, closed suction systems, and semi-recumbent positioning, were not standardized or not even practiced in many of the included studies. Therefore, current administration of antibiotics via the respiratory tract should be reevaluated in combination with such non-pharmacological preventive strategies. Most important of all, it cannot be overemphasized that no reduction in mortality was found between the compared groups in our meta-analysis. This is a noteworthy result that could be due to a sample size effect or, alternatively, due to lack of an effect of the administered preventive measure on mortality. However, even without a mortality benefit, the reduction of incidence of ICU-acquired pneumonia is associated with a reduction of length of ICU stay and costs.

## Conclusion

Despite the above limitations, we think that our study offers potentially useful data that may be of value to clinicians taking care of patients in the ICU setting. The relevant evidence from the available comparative trials shows that prophylactic administration of antibiotics via the respiratory tract in patients in the ICU setting is associated with reduction of occurrence of ICU-acquired pneumonia. However, it should be emphasized that evidence from non-comparative studies supports that this preventive strategy may lead to an increase in the emergence of resistant bacteria. We believe that the available evidence suggests that further investigation and consideration of this preventive strategy, including a more systematic evaluation of issues related to the emergence of resistance, is warranted, at least for ICU patients at high risk for development of ICU-acquired pneumonia.

## Key messages

• There is limited evidence regarding the role of administration of antimicrobial agents via the respiratory tract for the prevention of ICU-acquired pneumonia.

• Data from five RCTs included in our meta-analysis suggest that ICU-acquired pneumonia was less common in the group of patients that received antibiotic prophylaxis via the respiratory tract compared with those who received placebo or no therapy.

• No difference in mortality was found between the compared groups.

• Although there is evidence from non-comparative studies that this preventive strategy may lead to an increase in the emergence of resistant bacteria, data from the comparative trials included in our analysis do not allow us to comment on whether or not administration of topical antimicrobial agents in the respiratory tract is associated with the development of resistance.

## Abbreviations

CI = confidence interval; ICU = intensive care unit; OR = odds ratio; RCT = randomized controlled trial; VAP = ventilator-associated pneumonia.

## Competing interests

The authors declare that they have no competing interests.

## Authors' contributions

MEF had the idea, designed and supervised the study, and is the guarantor. IIS and IAB performed the literature search, identified the relevant studies to be included in the analysis, and extracted the data for the study. All authors contributed to the writing of the manuscript and approved its final version.

## Figures and Tables

**Table 1:** Characteristics of comparative trials included in the meta-analysis

Reference	Year	Type of trial	Study quality score	Study population/setting	Method for the micro-biological diagnosis of pneumonia	Length of ICU stay (days)	Duration of mechanical ventilation (days)	Studied drug/dosage	Drug administration	Mode of administration	ITT	No. of patients clinically evaluable
Wood *et al*. [21]	2002	Double-blind, placebo-controlled RCT	5	Mechanically ventilated for >2 days, trauma patients with >1 risk factor for post-traumatic pneumonia; ICU; USA	Bronchoalveolar lavage	19 ± 11 vs 21 ± 12	16 ± 11 vs 18 ± 13	Ceftazidime: 250 mg every 12 hours	For 7 days	Aerosolized	59	20 vs 20
Rouby *et al*. [19]	1994	Non-randomized clinical trial	NA	Mechanically ventilated for >3 days; surgical ICU; France	Bronchoalveolar lavage	No data	Survivors: 18 ± 12 vs 12 ± 14 Non-survivors: 9 ± 5 vs 8 ± 4	Colistin: 200,000 units every 3 h	For 2 weeks	Endotracheal instillation	598	347 vs 251
Rathgeber *et al*. [18]	1993	RCT	2	Mechanically ventilated; ICU; Germany	Bronchial secretions	No data	17 vs 13	Tobramycin: 80 mg every 6 hours	Until the time of extubation	Aerosolized	69	29 vs 40
Lode *et al*. [17]	1992	Double-blind, placebo-controlled, RCT	2	Mechanically ventilated for >3 days; 5 European ICUs	No data	No data	No data	Gentamicin: 40 mg every 6 hours	Until the time of extubation (<14 days)	Endotracheal instillation	199	85 vs 77
Vogel *et al*. [20]	1981	Non-randomized, controlled clinical trial	NA	Mechanically ventilated for >5 days; medical ICU; Germany	Tracheal aspirates	No data	8.3 vs 7.4	Gentamicin: 40 mg every 6 hours	For 2 weeks	Endotracheal instillation	40	20 vs 20
Klick *et al*. [15]	1975	Double-blind, placebo-controlled, non-randomized clinical trial	NA	Mechanically ventilated or not; respiratory-surgical ICU; USA	Sputum; Tracheal aspirates	5.1 vs 5.3	No data	Polymyxin B: 2.5 mg/kg body weight/day in 6 divided doses	Throughout the entire ICU stay	Aerosolized	744	355 vs 337
Klatersky *et al*. [13]	1974	Placebo-controlled RCT	3	Tracheostomised neurosurgical ICU; Belgium	Sputum; tracheal aspirates; bronchial secretions	19.9 vs 14.7	NA	Gentamicin: 80 mg every 8 hours	Throughout the entire ICU stay	Endotracheal instillation	110	43 vs 42
Greenfield *et al*. [11]	1973	RCT	1	Mechanically ventilated or not, high-risk patients; respiratory-surgical ICU; USA	Sputum	9.0 (median 6.0) vs 7.6 (median 6.0)	No data	Polymyxin B: 2.5 mg/kg body weight/day in 6 divided doses	Throughout the entire ICU stay	Aerosolized	58	33 vs 25

Values are for the group receiving prophylactic antibiotics by the respiratory tract versus (vs) the control group. ICU, intensive care unit; ITT, intention-to-treat; NA, non applicable; RCT, randomized controlled trial.

**Table 2:** Outcome data from the selected comparative trials for the meta-analysis

Reference	Year	ICU-acquired pneumonia (time of evaluation)	Mortality due to pneumonia (time of evaluation)	All cause mortality (time of evaluation)	Proportion of patients with colonization of respiratory tract by *P. aeruginosa*	Emergence of resistance	Toxicity
Wood *et al*. [21]	2002	3/20 (15%) vs 11/20 (55%) (day 14); 6/20 (30%) vs 13/20 (65%) (entire ICU stay)	NA	3/20 (15%) vs 6/20 (30%) (entire ICU stay)	No data	No clinically significant changes in bacterial sensitivity patterns^a^	None
Rouby *et al*. [19]	1994	97/347 (28%) vs 100/251 (40%) (week 2)	NA	42/347 (12%) vs 31/251 (12%) (week 2)	No data	Not observed^a^	Not mentioned
Rathgeber *et al*. [18]	1993	5/29 (17%) vs 17/40 (43%) (entire ICU stay)	2/29 (7%) vs 4/40 (10%) (entire ICU stay)	4/29 (14%) vs 8/40 (20%) (entire ICU stay)	2/171 (1%) vs 44/215 (20%)^b^	Non-significantly higher incidence mainly of *S. epidermidis*^a^	None
Lode *et al*. [17]	1992	29/85 (34%) vs 25/77 (32%) (day 16)	NA	23/85 (27%) vs 25/77 (39%) (week 4)	2/85 (2%) vs 6/77 (8%)	No data	Not mentioned
Vogel *et al*. [20]	1981	Less frequent in the gentamicin group	NA	No data	5/20 (25%) vs 9/20 (45%)	No evidence of increase^a^	Not mentioned
Klick *et al*. [15]	1975	16/355 (5%) vs 24/337 (7%) (entire ICU stay)	5/374 (1%) vs 2/370 (0.5%) (entire ICU stay)	45/374 (12%) vs 45/370 (12%) (entire ICU stay)	6/374 (2%) vs 36/370 (10%)	Did not occur to any significant extent^a^	Not mentioned
Klastersky *et al*. [13]	1974	5/43 (12%) vs 17/42 (40%) (entire ICU stay)	2/43 (5%) vs 4/42 (10%) (entire ICU stay)	23/43 (54%) vs 16/42 (38%) (entire ICU stay)	39/228 (17%) vs 32/174 (18%)^b^	The isolated microorganisms from the drug group were slightly more resistant to gentamicin^a^	Not mentioned
Greenfield *et al*. [11]	1973	2/33 (6%) vs 4/25 (16%) (entire ICU stay)	NA	4/33 (12%) vs 6/25 (24%) (entire ICU stay)	0/33 (0%) vs 3/25 (12%)	Not encountered frequently (only six Gram-negative bacteria resistant to polymyxin)^a^	Negligible^a^

Values are for the group receiving prophylactic antibiotics by the respiratory tract versus (vs) the control group. ^a^According to the investigators of the study. ^b^Refers to proportion of isolates. ICU, intensive care unit;
